# Channel Capacity of Concurrent Probabilistic Programs

**DOI:** 10.3390/e21090885

**Published:** 2019-09-12

**Authors:** Khayyam Salehi, Jaber Karimpour, Habib Izadkhah, Ayaz Isazadeh

**Affiliations:** Department of Computer Science, University of Tabriz, Tabriz 51666-16471, Iran

**Keywords:** channel capacity, information theory, evolutionary algorithms, quantitative information flow, concurrent probabilistic programs

## Abstract

Programs are under continuous attack for disclosing secret information, and defending against these attacks is becoming increasingly vital. An attractive approach for protection is to measure the amount of secret information that might leak to attackers. A fundamental issue in computing information leakage is that given a program and attackers with various knowledge of the secret information, what is the maximum amount of leakage of the program? This is called channel capacity. In this paper, two notions of capacity are defined for concurrent probabilistic programs using information theory. These definitions consider intermediate leakage and the scheduler effect. These capacities are computed by a constrained nonlinear optimization problem. Therefore, an evolutionary algorithm is proposed to compute the capacities. Single preference voting and dining cryptographers protocols are analyzed as case studies to show how the proposed approach can automatically compute the capacities. The results demonstrate that there are attackers who can learn the whole secret of both the single preference protocol and dining cryptographers protocol. The proposed evolutionary algorithm is a general approach for computing any type of capacity in any kind of program.

## 1. Introduction

Preventing leakage of secret information to public sources, accessible by attackers, is an important concern in information security. Quantitative information flow [1] is a well-established mechanism for measuring the amount of leakage occurred in a program. It has been successfully applied to many security applications, such as analyzing anonymity protocols [2,3] or the OpenSSL Heartbleed vulnerability [4].

An attacker, with prior knowledge of the secret information of the program, might execute the program and, based on the observation of public variables, infer further knowledge on the secrets (posterior knowledge). For example, in the program (l:=h mod 2), where l is a public output and h is a secret input, the attacker infers the rightmost bit of h by observing l; or in the program (l:=h&(110)${}_{b}$), where h is a 3-bit secret input and (110)${}_{b}$ is the 3-bit binary form of 6, the attacker learns the two leftmost bits of h. The attacker has an initial uncertainty about the secrets, which is the reverse of the prior knowledge and a remaining uncertainty, which is the reverse of the posterior knowledge. Then, information leakage is defined as

information leakage = initial uncertainty − final uncertainty.

Uncertainty can be quantified using information theory concepts, such as the Renyi’s min-entropy [1].

In analyzing concurrent probabilistic programs, the effect of the scheduler of the program should be considered, as different schedulers yield different amounts of leakage [5,6]. For example, consider the program (l:=1 || if l=1 then l:=h), where || is the concurrency operator with shared variables and the initial value of l is 0. If the attacker chooses a scheduler that runs (l:=1) first, then the amount of leakage would be 100%, but if they choose a scheduler that executes (if l=1 then l:=h) first, then the amount of leakage would be 0. It is also common to assume an attacker that can observe the public variables in every single step of the executions [6,7,8,9,10]. This observational power results in intermediate leakages. For example, in the program (l:=1 || if l=1 then l:=h); l:=0 with a scheduler that executes (l:=1) first, there is no leakage in the final step, but the whole bits of h get leaked in an intermediate step. Therefore, in analyzing concurrent probabilistic programs, leakage in intermediate steps and the effect of scheduler should be taken into account.

A frequently asked question in quantitative information flow is: what is the worst-case scenario? This wost-case leakage is called channel capacity, which is an upper bound of leakage over all attackers with the same observational power but different prior knowledge on the secrets [11]. Many approaches have been proposed to measure the capacity of programs. Most of these approaches model programs using channel matrices and compute the capacity using well-known mathematical techniques. For example, Malacaria and Chen [8] use Lagrange multipliers to compute the channel capacity. They use channel matrices to model various programs, including concurrent probabilistic ones. In another work [12], they use Karush–Kuhn–Tucker (KKT) to find the capacity. In using well-known mathematical techniques such as Lagrange multipliers and KKT to compute the capacity; it is necessary to prove the concavity of the leakage function. In most programs and leakage functions, concavity does not hold. Therefore, it is not always possible to use mathematical techniques for computing the capacity. Furthermore, channel matrices have exponential size [13] and are not suitable for considering intermediate leakages and scheduler effect [5,6].

In this paper we propose a fully-automated approach, which involves using Markovian processes for modeling programs and an evolutionary algorithm for computing the capacity. Assume a terminating concurrent program containing sequential probabilistic modules with shared variables. Furthermore, assume a probabilistic scheduler that determines the execution order of statements of the modules. Suppose an attacker that is capable of observing source code of the program, executing the program with a chosen scheduler, and observing the public variables in each step of the executions. The secret and the public variables are shared among the modules, but the attacker can only read the public variables. Following the approach introduced in our previous work [6], we use Markov chains to model executions of concurrent probabilistic programs under control of a probabilistic scheduler and compute expected and maximum leakages of a Markov chain. We then define two notions of capacity, $C_{E}$ and $C_{max}$, which are upper bounds of expected and maximum leakage over all possible prior knowledge of attackers. Computing these capacities is a constrained nonlinear optimization problem and concavity of the objective leakage function is not guaranteed. Thus, we propose a genetic algorithm to measure, approximately, the capacity values of $C_{E}$ and $C_{max}$. The algorithm has been implemented in Python. Finally, we discuss the anonymity protocols, the single preference voting, and the dining cryptographers as case studies to evaluate the proposed approach. We show how to apply the genetic algorithm to approximate the capacities for various cases of the protocols.

To the best of our knowledge, this is the first work in quantitative information flow that uses an evolutionary algorithm to compute the channel capacity and the first work that measures channel capacity for the single preference voting protocol.

In summary, our contributions are

- defining two notions of capacity, $C_{E}$ and $C_{max}$, for shared-memory concurrent probabilistic programs;
- a genetic algorithm for computing, approximately, the capacities $C_{E}$ and $C_{max}$;
- using the proposed approach for computing the capacities of the single preference voting protocol and the dining cryptographers protocol, which are upper bounds of anonymity leakage; and
- a formula to measure the capacities $C_{E}$ and $C_{max}$ of the single preference voting protocol in general.

The remaining of this paper is organized as follows. Section 2 reviews the related work. Preliminary concepts and Markovian processes are presented in Section 3. The information leakage and channel capacity of concurrent probabilistic programs are discussed in Section 4 and Section 5. The proposed evolutionary algorithm for computing the capacity of concurrent probabilistic programs is explained in Section 6. The case studies are discussed in Section 7. Finally, Section 8 concludes the paper and discusses future work.

## 2. Related Work

Channel capacity in the context of information security was introduced by Millen [14]. It has since gained attention and has been studied in many researches. Chatzikokolakis et al. [15] compute channel capacity of anonymity protocols that satisfy certain symmetries. They model the protocols using channel matrices. These matrices have been used in many quantitative works and are appropriate for analysis of sequential programs. However, their model is not suitable for computing intermediate and scheduler-specific leakages of concurrent probabilistic programs.

Inspired by the authors of [15], Malacaria and Chen use Lagrange multipliers to compute the channel capacity of programs with asymmetric matrix for different observational models of attackers [8]. In order to compute the capacity of a program, they manually derive the channel matrix of the program, consider a constrained nonlinear optimization problem, and solve a series of equations using Lagrange multipliers. In a further work by the authors of [2], they apply their approach to compute the capacity of anonymity protocols. In a further work by the authors of [12], they extend their approach using KKT conditions to support constraints expressed as inequality equations, which Lagrange multipliers are unable to handle. They have not implemented the approaches and have only demonstrated them using small examples. The concavity of the objective function in these examples are easily satisfied and thus they have used Lagrange multipliers and KKT to find the capacity. There  are many other programs, in which the concavity is not satisfied. Our approach is fully automatic and considers all variants of constraints.

Biondi et al. [11] measure the capacity of deterministic systems by interval Markov chains. They use the concept of entropy maximization in Bayesian statistics to maximize the entropy of a Markov model and compute the capacity. They only discuss final leakages of deterministic systems and do not consider the intermediate leakages.

Alvim et al. [16] define the additive and multiplicative notions of leakage and capacity based on any gain function. They also discuss computational aspects of measuring capacities. Chatzikokolakis [17] discuss more on the additive notion of leakage and capacity. In our proposed approach, we define capacity similar to the notion of multiplicative leakage with min-entropy. Alvim et al. state that in this case the existence of an efficient algorithm for computing the capacity is not certain. We develop a genetic algorithm to compute this type of capacity and evaluate the proposed algorithm using two anonymity protocols. The results show that the capacity values are not trivial.

Américo et al. [3] compute various types of channel capacities of two anonymity protocols. They define the capacities over all prior distributions and a fixed gain function, over a fixed prior distribution and all gain functions, etc. The Miracle theorem [18] proves that min-entropy is an upper bound of leakage over all gain functions. Therefore, we only established the capacity definitions over a gain function, i.e., Renyi’s min-entropy. Américo et al. use PCTL model checking of PRISM [19] to compute the various public outputs and their probabilities, by which the capacities are computed. They do not consider the intermediate leakages and specifying a formula using the PCTL logic requires considerable amount of manual effort.

An interesting point of view on information flow is side-channel attacks, in which the attacker can learn information about the secret values by observing the nonfunctional characteristics of program behavior [20]. Examples of side channels are computational time [21], power consumption [22] and cache behavior [23]. Side-channel attacks have been exploited in different situations, such as learning secret data from compression algorithms [24] or recovering cryptographic keys from an OpenSSL-based web server [25]. Protecting against side-channel attacks is hard to achieve. Malacaria et al. [20] propose symbolic side-channel analysis techniques to quantify information leakage for probabilistic programs. Kopf and Basin [26] quantify information leakage of adaptive side-channel attacks using information theory. Doychev et al. [27] discuss cache side-channel attacks for many attacker models. In our paper, we assume there is no side-channel attack on concurrent probabilistic programs and we formalize the channel capacity as well as propose an evolutionary algorithm to compute a near-optimum value for the capacity.

Another point of view on information flow is qualitative information flow, in which a security property is characterized to specify information flow requirements and a verification method is used to check satisfiability of the property. It has been studied well in the literature. Probabilistic noninterference [28,29,30] and observational determinism [10,31,32,33,34,35] have been used as information flow properties to characterize the security of concurrent programs. For verifying these security properties, type systems [28,29,31,32], algorithmic verification [10,30,33,34], program analysis [35], and logics  [36,37,38] have been utilized. In qualitative information flow, the security property gets rejected when there is a leakage, even a minor one. This is a restrictive condition and results in rejection of many trivially secure programs. For example, a password-checking program is rejected, because it reveals information on what the password is not. In quantitative information flow, the amount of leakage is quantified in order to allow minor leakages and reject major ones. Likewise, the channel capacity is quantified to determine an upper bound on the amount of leakage in the worst-case scenario.

## 3. Background

### 3.1. Information Theory

A probability distribution, Pr, over a set, Y, is a function, Pr:Y→[0,1], such that $\sum_{y\in Y}Pr\left(y\right)=1$. We denote the set of all probability distributions over Y by D(Y). Let X denote a random variable with the finite set of values $Val_{X}$ and the distribution $Pr\in D(Val_{X})$.

**Definition** **1.** *The **Renyi’s min-entropy** [1] of a random variable X is defined as*

$$H_{\infty }\left(X\right)=-\log_{2}\max_{x\in Val_{X}}\;Pr(x).$$

### 3.2. Markovian Models

We use Markov decision processes (MDPs) [39] to model operational semantics of concurrent probabilistic programs. MDPs model executions and traces of concurrent probabilistic programs using states and transitions. The concept of nondeterminism inherent in MDPs is used to model concurrency between modules by considering all possible choices of statements of the modules. We also use memoryless probabilistic schedulers, an important subclass of schedulers, to model the scheduling policy of the modules. A memoryless probabilistic scheduler resolves nondeterminism of MDPs and produces a Markov chain (MC), which only contains probabilistic transitions of the modules.

Suppose the program has a public output *l*. Formally,

**Definition** **2.** *A Markov decision process (MDP) is a tuple $M=(S,Act,P,\zeta ,Val_{l},V_{l})$ where,*

- *S is a set of states,*
- *Act is a set of actions,*
- *P:S→(Act→(S→[0,1])) is a transition probability function such that for all states s∈S and actions α∈Act:*
  $$\sum_{s^{\prime }\in S}P\left(s\right)\left(\alpha \right)\left(s^{\prime }\right)\in \{0,1\},$$
- *ζ:S→[0,1] is an initial distribution such that $\sum_{s\in S}\zeta \left(s\right)=1$,*
- *$Val_{l}$ is the finite set of values of l,*
- *$V_{l}:S\to Val_{l}$ is a labeling function.*

The function $V_{l}$ labels each state with value of the public variable in that state. In fact, a state label is what an attacker observes in a state. An MDP M is called finite if *S*, Act, and $Val_{l}$ are finite. An action α is enabled in state *s* if, and only if, $\sum_{s^{\prime }\in S}P\left(s\right)\left(\alpha \right)\left(s^{\prime }\right)=1$. Let Act(s) denote the set of enabled actions in *s*. Each state $s^{\prime }$ for which $P\left(s\right)\left(\alpha \right)\left(s^{\prime }\right)>0$ is called an *α-successor* of *s*. The set of α-successors of *s* is denoted by Post(s,α). The set of successors of *s* is defined as

$$Post\left(s\right)=\underset{\alpha \in Act(s)}{\cup }Post(s,\alpha ).$$

The state *s* with ζ(s)>0 is considered as an initial state. The set of initial states of M is denoted by Init(M). We assume the programs always terminate and thus all paths end in a state without any outgoing transition. We assume all blocking states correspond to the termination of the program. For technical reasons, we include a self-loop to each blocking state *s*, i.e., P(s)(τ)(s)=1, making all blocking states absorbing. A state *s* is called final if Post(s)={s}.

The intuitive operational behavior of an MDP M is as follows. At the beginning, an initial state $s_{0}$ is randomly chosen such that $\zeta (s_{0})>0$. Assuming that M is in state *s*, first a nondeterministic choice between the enabled actions needs to be resolved. Suppose action α∈Act(s) is selected. Then, one of the α-successors of *s* is selected randomly according to the transition function P. That is, with probability $P\left(s\right)\left(\alpha \right)\left(s^{\prime }\right)$ the next state is $s^{\prime }$.

An execution path of M is an infinite sequence of states that start in an initial state and loop infinitely in a final state. More precisely, a path is a state sequence $s_{0}s_{1}\ldots s_{n}^{\omega }$ such that $s_{i}\in Post\left(s_{i-1}\right)$ for all 0<i≤n, $s_{0}$ is initial and $s_{n}$ is final.

A trace of a path is the sequence of public values of the states of the path. Formally, the trace of an infinite path $\sigma =s_{0}s_{1}\ldots s_{n}^{\omega }$ is defined as $\bar{T}=trace\left(\sigma \right)=V_{l}\left(s_{0}\right)V_{l}\left(s_{1}\right)\ldots V_{l}{\left(s_{n}\right)}^{\omega }$. The set of traces of M is denoted by Traces(M). Let $Paths(\bar{T})$ be the set of paths that have the trace $\bar{T}$, i.e., $Paths\left(\bar{T}\right)=\left\{\sigma \;|\;\sigma \in Paths\left(M\right):trace\left(\sigma \right)=\bar{T}\right\}$.

**Definition** **3.** *A (discrete-time) Markov chain (MC) is a tuple $M=(S,P,\zeta ,Val_{l},V_{l})$ where,*

- *S is a set of states,*
- *P:S×S→[0,1] is a transition probability function such that for all states s∈S:*
  $$\sum_{s^{\prime }\in S}P\left(s,s^{\prime }\right)=1,$$
- *ζ:S→[0,1] is an initial distribution such that $\sum_{s\in S}\zeta \left(s\right)=1$,*
- *$Val_{l}$ is the finite set of values of l,*
- *$V_{l}:S\to Val_{l}$ is a labeling function.*

The function P determines for each state *s* the probability $P(s,s^{\prime })$ of a single transition from *s* to $s^{\prime }$. MCs are state transition systems with probability distributions for transitions of each state. That is, the next state is chosen probabilistically, not nondeterministically.

We define the occurrence probability of a trace $\bar{T}$ in an MC M as

$$Pr\left(T=\bar{T}\right)=\sum_{\sigma \in Paths(\bar{T})}Pr(\sigma ),$$

where *T* is a trace variable and

$$Pr\left(\sigma =s_{0}s_{1}\ldots s_{n}^{\omega }\right)=\left\{\begin{array}{c} \zeta (s_{0})\;\;\;\;\;\mathrm{if}\;n=0,\\ \zeta \left(s_{0}\right).\prod_{0\leq i<n}P\left(s_{i},s_{i+1}\right)\;\;\mathrm{otherwise}. \end{array}\right.$$

**Definition** **4.** *Let $M=(S,Act,P,\zeta ,Val_{l},V_{l})$ be an MDP. A memoryless probabilistic scheduler for M is a function δ:S→D(Act), such that δ(s)∈D(Act(s)) for all s∈S.*

As all nondeterministic choices in an MDP M are resolved by a scheduler δ, a Markov chain $M_{\delta }$ is induced. Formally,

**Definition** **5.** *Let $M=(S,Act,P,\zeta ,Val_{l},V_{l})$ be an MDP and δ:S→D(Act) be a memoryless probabilistic scheduler on M. The MC of M induced by δ is given by*

$$M_{\delta }=\left(S,P_{\delta },\zeta ,Val_{l},V_{l}\right)$$

*where*

$$P_{\delta }\left(s,s^{\prime }\right)=\sum_{\alpha \in Act(s)}\delta \left(s\right)\left(\alpha \right).P\left(s\right)\left(\alpha \right)\left(s^{\prime }\right)$$

## 4. Leakage of Concurrent Probabilistic Programs

In this section, we explain how to compute expected and maximum leakages of concurrent probabilistic programs, considering intermediate states. For further information, please see the work by the authors of [6].

Let P be a terminating concurrent probabilistic program and δ be a probabilistic scheduler. Suppose P has one public variable *l*, one secret variable *h*, and possibly several neutral variables. In case there are more public or secret variables, they can be encoded into one public or secret variable. Let $Val_{l}$ and $Val_{h}$ denote the finite sets of values of *l* and *h*, respectively, and Pr(h) denotes the attacker’s prior knowledge on the secret variable. Operational semantics of P is represented by an MDP $M^{P}=\left(S,Act,P,\zeta ,Val_{l},V_{l}\right)$, which models all possible interleavings of the modules. The scheduler is represented by a memoryless probabilistic scheduler δ. As the MDP $M^{P}$ is executed under the control of the scheduler δ, all nondeterministic transitions are resolved and an MC

$$M_{\delta }^{P}=\left(S,P_{\delta },\zeta ,Val_{l},V_{l}\right)$$

is produced. Each state of $M_{\delta }^{P}$ shows the current values of *h*, *l*, possible neutral variables, and the program counter. Since states of $M_{\delta }^{P}$ contain the program counter, loops of the programs are unfolded in $M_{\delta }^{P}$, and the programs always terminate, therefore $M_{\delta }^{P}$ contains no loops (ignoring self-loops of final states). It takes the form of a directed acyclic graph (DAG), with initial states as roots of the DAG and final states as leaves. Thus, reachability probabilities in $M_{\delta }^{P}$ coincide with long-run probabilities  [40].

As the attacker is able to observe the public values, the labeling function is restricted to *l*, i.e., $V_{l}:S\to Val_{l}$ and states are labeled by the value of *l* in the corresponding state. The initial distribution ζ is determined by the prior knowledge of the probabilistic attacker Pr(h). Let the function $V_{h}\left(s\right)$ determine the value of the variable *h* in the state s∈S. Then, $\zeta \left(s_{0}\right)=Pr\left(h=V_{h}\left(s_{0}\right)\right)$ for all $s_{0}\in Init\left(M_{\delta }^{P}\right)$.

The uncertainty of the attacker can be computed using either Shannon entropy, Renyi’s min-entropy, or any other gain function. Shannon entropy is used for attackers that guess the secret information in multiple tries, whereas Renyi’s min-entropy is a better measurement for computing uncertainty of attackers that guess the secret in only one try [1]. On the other hand, according to the Miracle theorem [18], Renyi’s min-entropy is an upper bound of leakage over all gain functions. Therefore, we measure the uncertainty of the attacker by the Renyi’s min-entropy.

The attacker’s initial knowledge is represented by the prior distribution Pr(h) and his final knowledge after executing the program and observing the traces is represented by the posterior distribution Pr(h|T). Therefore, the expected leakage of $M_{\delta }^{P}$ is computed as the difference of initial uncertainty and remaining uncertainty.

**Definition** **6.** *The expected leakage of the MC $M_{\delta }^{P}$ is computed as*

$$L_{E}\left(P_{\delta }\right)=H_{\infty }\left(h\right)-H_{\infty }\left(h|T\right),$$

*where $H_{\infty }\left(h\right)$ is the initial uncertainty and is computed as*

$$H_{\infty }\left(h\right)=-\log_{2}\max_{\bar{h}\in Val_{h}}Pr\left(h=\bar{h}\right)$$

*and $H_{\infty }\left(h|T\right)$ is the remaining uncertainty and is computed as*

$$H_{\infty }\left(h|T\right)=\sum_{\bar{T}\in Traces\left(M_{\delta }^{P}\right)}Pr\left(T=\bar{T}\right).H_{\infty }\left(h|T=\bar{T}\right),$$

*where $H_{\infty }\left(h|T=\bar{T}\right)$ is defined as*

$$H_{\infty }\left(h|T=\bar{T}\right)=-\log_{2}\max_{\bar{h}\in Val_{h}}Pr\left(h=\bar{h}|T=\bar{T}\right)$$

*and $Pr(h=\bar{h}|T=\bar{T})$ is computed by*

$$Pr\left(h=\bar{h}|T=\bar{T}\right)=\frac{Pr(h=\bar{h},T=\bar{T})}{Pr(T=\bar{T})}$$

*Pr(h,T) is the joint probability of h and T and is calculated by*

$$Pr\left(h=\bar{h},T=\bar{T}\right)=\sum_{\sigma \in Paths\left(\bar{T}\right),\;V_{h}\left(\sigma \left[0\right]\right)=\bar{h}}Pr\left(\sigma \right)$$

*where Pr(σ) is the occurrence probability of the path σ and $Pr(T=\bar{T})$ is the occurrence probability of the trace $\bar{T}$.*

Another measure for quantifying the security of a program is maximum leakage [6], which is the maximal value of leakages occurred in all execution traces of the program. Maximum leakage, denoted $L_{max}\left(P_{\delta }\right)$, is an upper bound of leakage that an attacker with prior knowledge Pr(h) can infer from $P_{\delta }$.

**Definition** **7.** *The maximum leakage of the MC $M_{\delta }^{P}$ is computed as*

$$L_{max}\left(P_{\delta }\right)=H_{\infty }\left(h\right)-\min_{\bar{T}\in Traces\left(M_{\delta }^{P}\right)}H_{\infty }\left(h|T=\bar{T}\right).$$

## 5. Capacity of Concurrent Probabilistic Programs

Capacity is an upper bound of leakage over all possible distributions of the secret input. We consider two types of leakages, expected and maximum. Therefore, two types of capacities are defined: $C_{E}$ and $C_{max}$. Formally,

**Definition** **8.** *The* capacity*$C_{E}$ of the MC $M_{\delta }^{P}$ is defined as*

$$\begin{array}{cc} C_{E}\left(P_{\delta }\right) & =\max_{Pr\left(h\right)\in D\left(Val_{h}\right)}L_{E}\left(P_{\delta }\right)\\ \; & =\max_{Pr\left(h\right)\in D\left(Val_{h}\right)}\left(H_{\infty }\left(h\right)-\sum_{\bar{T}\in Traces\left(M_{\delta }^{P}\right)}Pr\left(T=\bar{T}\right).H_{\infty }\left(h|T=\bar{T}\right)\right) \end{array}$$

*where $H_{\infty }\left(h\right)$, Pr(T), and $H_{\infty }\left(h|T=\bar{T}\right)$ depend on Pr(h).*

**Definition** **9.** *The* capacity*$C_{max}$ of the MC $M_{\delta }^{P}$ is defined as*

$$\begin{array}{cc} C_{max}\left(P_{\delta }\right) & =\max_{Pr\left(h\right)\in D\left(Val_{h}\right)}L_{max}\left(P_{\delta }\right)\\ \; & =\max_{Pr\left(h\right)\in D\left(Val_{h}\right)}\left(H_{\infty }\left(h\right)-\min_{\bar{T}\in Traces\left(M_{\delta }^{P}\right)}H_{\infty }\left(h\right|T=\bar{T}\right) \end{array}$$

*where $H_{\infty }\left(h\right)$, Pr(T), and $H_{\infty }\left(h|T=\bar{T}\right)$ depend on Pr(h).*

Note that for every program we have $C_{E}\leq C_{max}$.

**Example** **1.** *Consider the following program P1*.

    l:=0;
    l:=h/2 || l:=h mod 2

*where h is the secret input with $Val_{h}=\left\{0,1,2\right\}$, l is the public output, and || is the parallel operator.*

**Figure 1:**
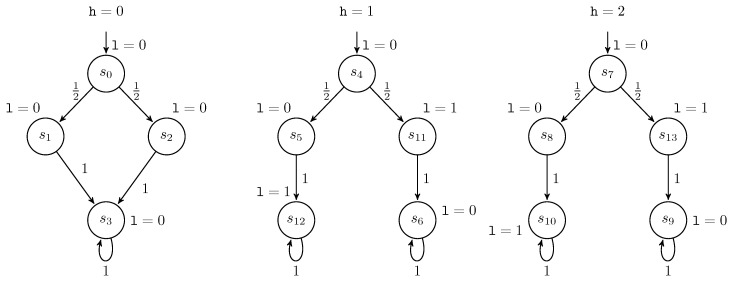
$M_{uni}^{P1}$: MC of the program P with the uniform scheduler.

*The Markov chain $M_{uni}^{P1}$ of the program, running under control of a uniform scheduler uni, is depicted in Figure 1.*

*In this MC, each state is labeled by the value of l in that state and each transition is labeled by a probability. For instance, the transition from $s_{0}$ to $s_{1}$ has the probability $P_{uni}\left(s_{0},s_{1}\right)=\frac{1}{2}$.*

*Assume the attacker’s prior knowledge over the secret variable h is*

$$Pr\left(h\right)=\left\{0\mapsto p_{0},\;1\mapsto P_{1},\;2\mapsto p_{2}\right\}$$

*where $p_{i}$ is the probability of choosing h=i. The initial uncertainty is quantified as the Renyi’s min-entropy of h in the initial states:*

$$\mathrm{initial}\;\mathrm{uncertainty}=H_{\infty }\left(h\right)=-\log_{2}\max_{i\in \{0,1,2\}}p_{i}$$

*The remaining uncertainty is quantified as the Renyi’s min-entropy of h after observing the traces. There are three traces with different occurrence probabilities:*

$$\begin{array}{c} T_{0}=<0,0,0^{\omega }>,\;Pr\left(T=T_{0}\right)=p_{0},\\ T_{1}=<0,0,1^{\omega }>,\;Pr\left(T=T_{1}\right)=\frac{1}{2}\left(p_{1}+p_{2}\right),\\ T_{2}=<0,1,0^{\omega }>,\;Pr\left(T=T_{2}\right)=\frac{1}{2}\left(p_{1}+p_{2}\right). \end{array}$$

*Each trace results in different sets of final states, with different posterior distributions:*

$$\begin{array}{c} Pr\left(h|T=T_{0}\right)=\left\{0\mapsto 1\right\},\\ Pr\left(h|T=T_{1}\right)=\left\{1\mapsto \frac{p_{1}}{p_{1}+p_{2}},2\mapsto \frac{p_{2}}{p_{1}+p_{2}}\right\},\\ Pr\left(h|T=T_{2}\right)=\left\{1\mapsto \frac{p_{1}}{p_{1}+p_{2}},2\mapsto \frac{p_{2}}{p_{1}+p_{2}}\right\}. \end{array}$$

*Consequently, the remaining uncertainty is quantified as*

$$\begin{array}{cc} remaining\;uncertainty & =\sum_{i\in \{0,1,2\}}Pr\left(T_{i}\right).H_{\infty }\left(h|T=T_{i}\right)\\ & =-\left(p_{1}+p_{2}\right)*\log_{2}\max\left\{\frac{p_{1}}{p_{1}+p_{2}},\frac{p_{2}}{p_{1}+p_{2}}\right\} \end{array}$$

*and the expected leakage of the program ${P1}_{uni}$ is computed as*

$$\begin{array}{c} \;\;L_{E}\left({P1}_{uni}\right)=\left(-\log_{2}\max_{i\in \{0,1,2\}}p_{i}\right)+\left(p_{1}+p_{2}\right)*\log_{2}\max\left\{\frac{p_{1}}{p_{1}+p_{2}},\frac{p_{2}}{p_{1}+p_{2}}\right\}. \end{array}$$

*These yield the capacity $C_{E}$ of the program ${P1}_{uni}$ is computed as*

$$\begin{array}{cc} C_{E}\left({P1}_{uni}\right)= & \max_{p_{i}}\left(\left(-\log_{2}\max_{i\in \{0,1,2\}}p_{i}\right)+\left(p_{1}+p_{2}\right)*\log_{2}\max\left\{\frac{p_{1}}{p_{1}+p_{2}},\frac{p_{2}}{p_{1}+p_{2}}\right\}\right)\\ \; & subject\;to\;\;\;\sum_{i\in \{0,1,2\}}p_{i}=1. \end{array}$$

*Now, we explain how to compute the capacity $C_{max}$ of ${P1}_{uni}$. Since the minimum Renyi’s min-entropy in the final states is 0, then the maximum leakage of ${P1}_{uni}$ is*

$$\begin{array}{c} \;\;L_{max}\left({P1}_{uni}\right)=-\log_{2}\max_{i\in \{0,1,2\}}p_{i}\;, \end{array}$$

*and the capacity $C_{max}$ of ${P1}_{uni}$ is computed as*

$$\begin{array}{c} C_{max}\left({P1}_{uni}\right)=\max_{p_{i}}\left(-\log_{2}\max_{i\in \{0,1,2\}}p_{i}\right)\;\;\;\;subject\;to\;\;\;\sum_{i\in \{0,1,2\}}p_{i}=1. \end{array}$$

*Now, suppose the attacker’s prior knowledge is a uniform distribution on h*

$$Pr\left(h\right)=\left\{0\mapsto \frac{1}{3},\;1\mapsto \frac{1}{3},\;2\mapsto \frac{1}{3}\right\}$$

*i.e., $p_{i}=\frac{1}{3},\;\;i=0,1,2$. Then, the expected and the maximum leakages of ${P1}_{uni}$ are computed as*

$$\begin{array}{cc} \; & L_{E}\left({P1}_{uni}\right)=1.585-0.667=0.918\;\left(bits\right),\\ \; & L_{max}\left({P1}_{uni}\right)=1.585\;\left(bits\right), \end{array}$$

*whereas, for the distribution*

$$Pr\left(h\right)=\left\{0\mapsto \frac{1}{4},\;1\mapsto \frac{1}{2},\;2\mapsto \frac{1}{4}\right\}$$

*on h, we have*

$$\begin{array}{cc} \; & L_{E}\left({P1}_{uni}\right)=1-0.439=0.561\;\left(bits\right),\\ \; & L_{max}\left({P1}_{uni}\right)=1\;\left(bit\right). \end{array}$$

*In order to compute the capacities $C_{E}\left(P1\right)$ and $C_{max}\left(P1\right)$, we use the proposed evolutionary algorithm of Section 6. The capacity $C_{E}$ of P1 is 1 bit and is achieved by the distribution*

$$Pr\left(h\right)=\left\{0\mapsto \frac{1}{2},\;1\mapsto 0,\;2\mapsto \frac{1}{2}\right\}$$

*on h. On the other hand, the capacity $C_{max}$ of P1 is 1.585 bits and is achieved by the uniform distribution on h.*

**Figure 2:**
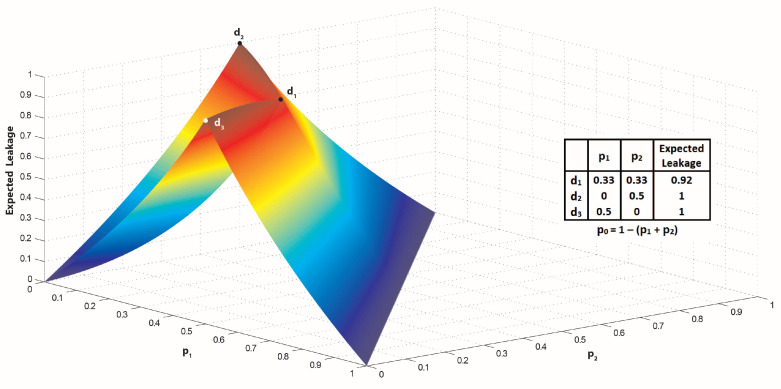
The expected leakage of ${P1}_{uni}$ ($L_{E}\left({P1}_{uni}\right)$).

Computing the capacities $C_{E}$ and $C_{max}$ is a constrained nonlinear optimization problem. The objective function for the problem is expected or maximum leakage of $M_{\delta }^{P}$. Recall that the leakage functions are computed as the difference of initial and remaining uncertainties and the uncertainties are computed using the Renyi’s min-entropy. The concavity of the objective functions is not necessarily satisfied. For example, Figure 2 depicts the expected leakage function of ${P1}_{uni}$ for various values of $p_{0}$, $p_{1}$ and $p_{2}$.

As demonstrated in this figure, the channel capacity for the expected leakage is 1 bit and occurs in the distributions $d_{2}=\left\{0\mapsto 0.5,1\mapsto 0,2\mapsto 0.5\right\}$ and $d_{3}=\left\{0\mapsto 0.5,1\mapsto 0.5,2\mapsto 0\right\}$. An interesting point in the figure is that the uniform distribution $d_{1}$ on *h* is not the channel capacity.

Figure 2 shows that the expected leakage function $L_{E}\left({P1}_{uni}\right)$ is not concave. Therefore, well-known mathematical techniques such as Lagrange multipliers [41,42] and Karush–Kuhn–Tucker (KKT) [42] cannot be used for optimizing the objective functions of $C_{E}$ and $C_{max}$. In this paper, a genetic algorithm is proposed to compute, approximately, the channel capacity of the concurrent probabilistic programs.

## 6. An Evolutionary Algorithm for Computing Capacity

The evolutionary algorithm proposed for computing near-optimum values for the two types of capacities is shown in Algorithm 1.

Problem space. The problem space is the probability space of the secret values. That is, for the i-th value of a secret variable, $p_{i}$ defines the probability of the attacker choosing that value. Note that sum of the probabilities must be equal to 1.

Coding method. The chromosome structure should contain sufficient information about the probability space. Hence, considering a vector of bits to encode a probability space is sufficient. In classical genetic algorithms, the chromosome structure consists of bits. In this paper for each probability of the secret values, a string of ten bits is considered. Therefore, the chromosome size is equal to $10*|Val_{h}|$.

Initial population. The set of chromosomes is called a population. The initial population is randomly generated by a uniform distribution. The number of chromosomes in the population and the maximum number of generations are set to 1500 and 2000, respectively.

Fitness function. During each generation, chromosomes are evaluated using the fitness function. For the evaluation, each chromosome is converted to a value between 0 and 1 to represent a probability value. For example, the chromosome “0100100011” is considered as the binary number 0.0100100011, and then converted to the probability 0.284. Based on these probability values, the expected leakage for $C_{E}$ or the maximum leakage for $C_{max}$ is computed using Definitions 6 or 7, respectively. Note that sum of the probabilities should be equal to 1. This is a constraint to the fitness function. In this situation, a penalty can be used for the fitness values of those chromosomes that do not satisfy the constraint [43]. We considered the absolute difference between the sum of the probabilities of a chromosome and 1 as a penalty, and is subtracted from the fitness value of the chromosome.

Selection, crossover, and mutation. In this paper, the roulette wheel method [44] is used to select chromosomes for mating (crossover) and producing new offspring. Chromosomes with higher fitness value have higher probability of mating and chromosomes with lower fitness value have lower probability of mating. After selection, the single-point crossover is used to mate the selected parents with probability $p_{c}$ (line 5 of Algorithm 1). In the single-point crossover, a point is chosen randomly and bits to the right of the chosen point are swapped between the two parent chromosomes. Thus, some genetic information from both parents are carried to the new offspring. To maintain the genetic diversity from one generation to the next and to avoid local minimum, the resulted offspring is mutated with probability $p_{m}$ (line 6 of Algorithm 1). In the mutation process, a point in the resulted offspring’s chromosome is picked randomly and the bit in that position is flipped.

**Algorithm 1** An evolutionary algorithm for computing capacity

**Input:**
       The Markov chain $M_{\delta }^{P}$       $p_{c}$: Crossover probability       $p_{m}$: Permutation probability       n_generations: Number of generations   
**Output:**
       The capacity $C_{E}$ or $C_{max}$ of the concurrent probabilistic program $P_{\delta }$
1:Initialize the chromosomes; // *initial population*2:Evaluate the fitness of each chromosome (candidate solution) using $M_{\delta }^{P}$;3:
**for** *i* **in** n_generations **do**
4:    parents = Select the fittest chromosomes for the next generation;5:    offspring = Mate the pairs of selected parents with the probability $p_{c}$;6:    offspring = Mutate the resulted offspring with the probability $p_{m}$;7:    Evaluate the fitness of the generated offspring using $M_{\delta }^{P}$;8:    Replace the offspring in the population considering the elitism;9:
**end for**
10:**return** best fitness of the population as capacity;


## 7. Implementation and Case Studies

As case study, two anonymous protocols, the single preference voting protocol [45] and the dining cryptographers protocol [46] are discussed. We show how to apply the proposed genetic algorithm to approximately compute the capacities for different cases of these protocols.

We used the PRISM language [19] to implement the case studies and the PRISM-Leak tool [6] to build the Markov model of the programs and extract the set of traces and their probabilities. These traces and probabilities were given as input to the genetic algorithm to compute the capacity values. The PRISM source codes of the protocols and the genetic algorithm are publicly available from the work by the authors of [47].

### 7.1. Case Study A: The Single Preference Voting Protocol

Assume a voting protocol with *c* candidates, *n* voters, and a winner (with majority votes). Each voter expresses a single preference to one of the candidates. Then, votes of each candidate are summed up and the candidate with the most votes wins the voting. In order to preserve the anonymity of the voters, only the counting results are publicly announced. Therefore, votes are secret and counting results are public. Votes and results are encoded into a single secret variable and a single public variable. There are $c^{n}$ secret values and thus the secret variable has a size of $n\log_{2}c$ bits.

The capacity values computed for the single preference protocol are shown in Table 1. In this table, $L_{E}$ denotes the expected leakage (Definition 6) and $Pr_{uni}\left(h\right)$ shows the the uniform distribution on *h*. The percentages are computed as the amount of leakage over the initial uncertainty.

As expected, in all cases of Table 1, $C_{max}$ is greater than or equal to $C_{E}$, and both capacity values are greater than the expected leakage with uniform distribution. However, the percentages for the capacities are all 100% and the votes get completely leaked.

Consider the case where c=2 and n=2. In this case, the secret values are “1-1”, “2-2”, “1-2”, and “2-1”. The secret value “1-2” means that the first voter has chosen the candidate 1 and the second one has picked the candidate 2. The secret size is $n\log_{2}c=2$ bits. An attacker that only knows the number of candidates and the number of voters, i.e., a uniform distribution on the secret values, observes four different traces. Each of the secret values “1-1” and “2-2” results in just one trace, and thus the attacker can infer the whole secret (who voted whom) by observing the corresponding trace. Both secret values “1-2” and “2-1” result in two traces and hence the attacker has to guess the secret value by a success probability of 50%. Therefore, the expected leakage of the single preference protocol for c=2 and n=2 becomes 1.5 and the maximum leakage becomes 2. The latter occurs because there are two traces that leak the whole secret (traces of “1-1” and “2-2”). Now consider an attacker that knows the secret values belong to {“1-1”, “2-2”, “2-1” }, which results in the prior distribution $\left\{‘‘1-1^{\prime \prime }\mapsto \frac{1}{3},\;‘‘2-2^{\prime \prime }\mapsto \frac{1}{3},\;‘‘2-1^{\prime \prime }\mapsto \frac{1}{3}\right\}$. When this attacker executes the program of the single preference protocol, they observe only one trace for each secret value and can infer each secret value by observing its corresponding trace. Therefore, the expected leakage for this attacker is equal to the whole secret size, that is, $\log_{2}3=1.58\;\left(100\%\right)$. The other attackers with the same observational power but different prior knowledge on the secrets infer less or equal information than the latter attacker. Thus, $C_{E}$ for c=2 and n=2 is 1.58 bits. Since the maximum leakage is 2(100%), it is clear that $C_{max}$ would be 2(100%), too.

**Lemma** **1.** *The capacity $C_{E}$ for the single preference protocol with n voters and c candidates corresponds to*

log2n+c−1n.

**Proof.**  All permutations of the same set of vote values produce the same set of traces. For instance for c=3 and n=2, there are nine secret values: “1-1”, “2-2”, “3-3”, “1-2”, “2-1”, “1-3”, “3-1”, “2-3”, and “3-2”. The secret value “1-3” means that the first voter has chosen the candidate 1 and the second one has picked the candidate 3. The secret values “1-3” and “3-1” produce the same set of traces and the attacker cannot distinguish between the traces. Thus, the secret values “1-3” and “3-1” form an equivalence class (a combination of vote values). Due to the fact that the maximum value of min-entropy is achieved by a uniform distribution, to compute the channel capacity of the program, it is sufficient to choose only one permutation of the secret values from each class and assign an equal probability to the selected classes. Since, the number of classes is equal to the number of the combinations of *c* candidates in *n* places with repetitions, and the selected secret values have the same probability, the channel capacity for the expected leakage corresponds to

log2n+c−1n.

 □

For c=3 and n=2, there are six equivalence classes of the votes: “1-1”, “2-2”, “3-3”, “1-2”, “1-3”, and “2-3”. A distribution that assigns $\frac{1}{6}$ to each class and 0 to the other secret values, i.e., “2-1”, “3-1”, and “3-2”, leads to the value 2.58 for $C_{E}$.

**Lemma** **2.** *The capacity $C_{max}$ for the single preference protocol with n voters and c candidates corresponds to*

$$n\log_{2}c,$$

*which is achieved by a uniform distribution on the secret values.*

**Proof.**  There is only one trace for each combination of the same vote values. For instance, for the case of c=3 and n=2, there is one trace for each of “1-1”, “2-2”, and “3-3”, and the attacker can learn the whole secret value by observing the trace. Thus, the remaining Renyi’s min-entropy of these traces is 0. To compute the channel capacity for the maximum leakage, it is enough to maximize the initial Renyi’s min-entropy. This is obtained by a uniform distribution on all secret values and the channel capacity is equal to $\log_{2}$ of the secret size, i.e., $n\log_{2}c$. □

### 7.2. Case Study B: The Dining Cryptographers Protocol

The dining cryptographers protocol [46] is an anonymous broadcasting protocol. In this protocol, a number of cryptographers are sitting around a round table to have dinner. They are informed that the dinner has been paid by either one of them or their master. The cryptographers intend to understand whether the master is paying or not. To solve the problem, each cryptographer tosses an unbiased coin and shows the result to his right side cryptographer. Then, he announces 1 if his coin and his left side cryptographer’s coin is the same or 0 if not. Of course, if the cryptographer is the payer, he lies and announces the inverse. To understand whether the payer is master or not, XOR of all announcements is computed. For an even number of cryptographers, a result of 1 implies that the master is the payer (none of the cryptographers is the payer) and a result of 0 shows that one of the cryptographers is the payer. This is reverse for an odd number of cryptographers.

Suppose an attacker who aims to infer the identity of the payer. The attacker can be

- internal, i.e., one of the cryptographers, who can see his own coin, the left side cryptographer’s coin, and also the announcements of all cryptographers, or
- external, i.e., none of the cryptographers or master, who can see the announcements of all cryptographers and XOR of the announcements;

and the payer can be

- one of the cryptographers, i.e., $Val_{payer}=\left\{c_{1},\ldots ,c_{N}\right\}$,
- the master (*m*, for short) or one of the cryptographers, i.e., $Val_{payer}=\left\{m,c_{1},\ldots ,c_{N}\right\}$.

The capacity values for the external attacker and the internal attacker with uniform scheduler are shown in Table 2 and Table 3, respectively. In these tables, *N* denotes the number of cryptographers and $\mu_{uni}$ shows the uniform distribution on *h*. In all cases of Table 2 and Table 3, $C_{max}$ is greater than or equal to $C_{E}$.

In the last three cases of Table 2, the leakage and capacity values are all 0. This demonstrates that when the master is not a payer candidate, the payer can not be recognized by the external attacker and the dining cryptographers protocol is secure. Another interesting point is that in both Table 2 and Table 3, except the last three rows of Table 2, $C_{max}$ is equal to $\log_{2}\left|Val_{payer}\right|$, i.e., 100% leakage. This demonstrates that the attacker identifies the payer.

In the first three cases of Table 2, where the attacker is external and $Val_{payer}=\left\{m,c_{1},\cdots ,c_{N}\right\}$, $C_{E}$ is equal to 1 bit for all values of *N*. This is achieved by a probability distribution, in which the probabilities of the payer being master and one of the cryptographers are equal to 50% and other probabilities are equal to 0. Likewise, in the last three rows of Table 3, where the attacker is internal and the payer is one of the cryptographers, $C_{E}$ is also 1 bit for all values of *N*. This is achieved by a distribution, in which the probabilities of two cryptographers are equal to 50% and the others are 0. Furthermore, in all cases that the attacker is internal and $Val_{payer}=\left\{m,c_{1},\cdots ,c_{N}\right\}$, the capacity $C_{E}$ is equal to 1.58. This is the result of a probability distribution, in which the probabilities of the master and two of the cryptographers are equal to $\frac{1}{3}$ and the others 0. This demonstrates that the internal attacker identifies the payer.

Stability evaluation. The evolutionary algorithms, including genetic algorithms, are meta-heuristic optimizers. Therefore, to rely on the results, the algorithm should be run multiple times and stability of the results be evaluated. The stability is defined as closeness of the results to each other in various runs. To evaluate the stability, *t*-test and Levene’s test [48], two well-known statistical techniques, are used. The Levene’s test investigates the equal variance assumption and based on its results, *t*-test verifies the equality of means of two independent groups of results.

For the evaluation, the proposed genetic algorithm was executed 30 times and the capacity $C_{E}$ was computed for a single case, i.e., internal attacker, N=3 and $Val_{payer}=\left\{c_{1},c_{2},c_{3}\right\}$. The $C_{E}$ and this case were chosen as an example of all possible capacities and cases. The results were divided into two groups of 15 runs.

The stability test for the experimental results of the dining cryptographers protocol is demonstrated in Table 4.

Since the significant value (Sig.) of the Levene’s test for equality of variance (0.207) is greater than the significance level 0.01, the equal variance assumption is accepted and the first row of *t*-test is considered. The significance level (0.235) is greater than 0.01 and the interval of the difference between means contains 0; it follows that with a confidence of 99% there are no statistically significant differences between means of the results in the groups. Thus, the algorithm results are stable in the sense that the number of runs is enough.

## 8. Conclusions and Future Work

In this paper, we discussed how to compute the channel capacity of concurrent probabilistic programs. We modeled the programs via Markovian processes and defined two types of capacities, which are upper bounds on expected and maximum leakages. These capacities range over all prior distributions of the secret. We proposed a genetic algorithm to approximate the capacity values. To show the applicability and feasibility of the proposed approach, the single preference voting and the dining cryptographers protocols were discussed. A *t*-test was performed to evaluate the stability of results produced by multiple runs of the evolutionary algorithm for the dining cryptographers protocol.

Our definition of capacities were of type multiplicative. As future work, we aim to define additive variants of capacity, ranging over all gain functions and all initial distributions. We plan to apply the proposed genetic algorithm to approximate these different types of capacities. As another future work, we will analyze other anonymity protocols, such as crowds and TOR, to observe how these protocols leak sensitive information in the worst-case scenarios. An interesting future work would be to compute the capacity of nonterminating concurrent probabilistic programs.

## Figures and Tables

**Table 1:** Capacity values in bits for the single preference protocol.

*c*	*n*	$L_{E}$ with ${\mathrm{Pr}}_{\mathrm{uni}}\left(h\right)$	$C_{E}$	$C_{\max}$
2	2	1.5 (75%)	1.58 (100%)	2 (100%)
	3	1.81 (60%)	2 (100%)	3 (100%)
3	2	2.5 (78%)	2.58 (100%)	3.17 (100%)
	3	3.12 (65%)	3.33 (100%)	4.75 (100%)
4	2	3.25 (81%)	3.33 (100%)	4 (100%)
	3	4.14 (69%)	4.33 (100%)	6 (100%)

**Table 2:** Capacity values in bits for the external attacker of the dining cryptographers protocol.

${\mathrm{Val}}_{\mathrm{payer}}$	*N*	$L_{E}$ with $\mu_{\mathrm{uni}}$	$C_{E}$	$C_{\max}$
$\left\{m,c_{1},\ldots ,c_{N}\right\}$	3	0.811 (40%)	1 (100%)	2 (100%)
	4	0.721 (31%)	1 (100%)	2.32 (100%)
	5	0.65 (25%)	1 (100%)	2.58 (100%)
$\left\{c_{1},\ldots ,c_{N}\right\}$	3	0	0	0
	4	0	0	0
	5	0	0	0

**Table 3:** Capacity values in bits for the internal attacker of the dining cryptographers protocol.

${\mathrm{Val}}_{\mathrm{payer}}$	*N*	$L_{E}$ with $\mu_{\mathrm{uni}}$	$C_{E}$	$C_{\max}$
$\left\{m,c_{1},\ldots ,c_{N}\right\}$	3	1.5 (75%)	1.58 (100%)	2 (100%)
	4	1.37 (59%)	1.58 (100%)	2.32 (100%)
	5	1.25 (48%)	1.58 (100%)	2.58 (100%)
$\left\{c_{1},\ldots ,c_{N}\right\}$	3	0.918 (58%)	1 (100%)	1.58 (100%)
	4	0.811 (40%)	1 (100%)	2(100%)
	5	0.72 (31%)	1 (100%)	2.32 (100%)

**Table 4:** The stability test of the experimental results for the significance level of 0.01.

		Levene’s Test for Equality of Variances	T-test for Equality of Means		
		Sig.	Sig. (2-tailed)	99% Confidence Interval of the Difference	
				Lower	Upper
Fitness	Equal variances assumed	0.207	0.235	−0.008736	0.003403
	Equal variances not assumed	-	0.235	−0.00874	0.003410
